# Importance of and Satisfaction with Domains of Health-Related Quality of Life in Cancer Rehabilitation

**DOI:** 10.3390/cancers14081991

**Published:** 2022-04-14

**Authors:** Andreas Hinz, Thomas Schulte, Jochen Ernst, Anja Mehnert-Theuerkauf

**Affiliations:** 1 Department of Medical Psychology and Medical Sociology, University of Leipzig, 04103 Leipzig, Germany; jochen.ernst@medizin.uni-leipzig.de (J.E.); anja.mehnert@medizin.uni-leipzig.de (A.M.-T.); 2 Rehabilitation Clinic Bad Oexen, 32549 Bad Oeynhausen, Germany; tschulte@badoexen.de

**Keywords:** quality of life, cancer, rehabilitation, importance, satisfaction

## Abstract

**Simple Summary:**

Quality of life (QoL) has gained increasing importance in oncology in general, and in cancer rehabilitation in particular. Multiple instruments have been developed to measure QoL. These instruments generally comprise several aspects of QoL, but they do not consider the subjective importance of these aspects. In our study, we assess the satisfaction with such aspects of QoL and the subjective importance of these aspects as well, based on a large sample of participants of a cancer rehabilitation program. The main result was that the subjective importance of domains of QoL is only weakly correlated with the detriments in these dimensions and that health care professionals should also consider what importance the patients attribute to these dimensions of QoL.

**Abstract:**

Instruments for measuring health-related quality of life (HRQoL) generally do not consider the subjective importance of the dimensions they comprise. The aims of this study were to analyze the subjectively perceived importance of the dimension of HRQoL and to investigate their relationship to the satisfaction ratings with these dimensions. A total of 1108 participants enrolled in a cancer rehabilitation program were surveyed. Patients rated eight dimensions of HRQoL (physical functioning, autonomy, emotional stability, cognitive functioning, social relationships, vitality, absence of pain, and sleep quality), as well as global health in terms of how important those dimensions are to them, and how satisfied they are with them. The dimensions with the highest importance ratings were autonomy and social relationships. There were only small sex differences in the importance ratings, but younger patients rated health as being more important than older patients did. The correlations between the importance ratings and the satisfaction ratings of the specific HRQoL dimensions ranged from −0.06 to 0.40, and the correlation between importance and satisfaction for global health was 0.01. Importance ratings provide relevant information for health care professionals in addition to the HRQoL assessments in the context of cancer rehabilitation.

## 1. Introduction

Health-related quality of life (HRQoL) has gained increasing importance in oncology [1,2]. Multiple instruments for measuring HRQoL have been developed. The article by Lehmann et al. on this issue [3] provides a very comprehensive and up-to-date overview of these instruments, in particular with regard to their use in cancer rehabilitation. The instruments generally cover several domains of HRQoL assumed to be relevant for most people. However, it is obvious that the different dimensions of HRQoL do not have the same meaning and importance for each person. This is specifically relevant in cases of severe diseases and in the palliative setting. Attempts have been made to qualify the HRQoL assessments by considering the subjective importance of the dimensions as weighting factors [4,5]; examples of these attempts are the Schedule for the Evaluation of Individual Quality of Life (SEIQoL) [6] and the Patient Generated Index (PGI) [7]. One severe disadvantage of these instruments is that each patient rates different components of QoL, and it is problematic to compare the results obtained by different persons. To conduct standardized and generalizable statistical analyses, it is more advisable to present a fixed set of dimensions to the patients, and then ask them to evaluate these dimensions concerning their subjective importance to them and their satisfaction with them. This technique has been adopted in several sociological studies that tested whether the inclusion of importance ratings improved the general assessment of QoL [8,9,10,11,12].

A further relevant research question concerns the degree to which the subjective importance of the dimensions is associated with the satisfaction experienced in these dimensions. One might assume that for dimensions such as health, low scores in satisfaction, e.g., the perception of health problems, would lead to an increase in subjective importance and that this effect would result in a negative correlation between importance and satisfaction ratings. However, several studies have shown that this is not the case. General population studies even found positive correlations between health importance and health satisfaction [8,11,13,14]. Since these studies included health as one global dimension of QoL without considering separate facets of HRQoL, they do not provide a more detailed insight into the components of HRQoL. One central aim of our study was to analyze the relationship between importance and satisfaction for several components of HRQoL in cancer patients.

The subjective importance of dimensions of HRQoL may depend on sex and age. General population studies have shown that satisfaction with health decreases with increasing age in the general population, while the importance of health increases [15,16]. In our study, we intend to test whether this is also the case for cancer patients in a rehabilitation setting, and which dimensions of HRQoL show specific sex and age effects.

The importance of an HRQoL dimension can be assessed in two ways. The first way entails using the direct importance ratings as indicated above. The second way is to infer to what degree the single HRQoL dimension contributes to the general assessment of overall QoL. This contribution can be expressed in terms of correlation or regression coefficients [8]. For example, in a sample of urologic cancer patients, the mean importance rating of the health dimension was the highest of the eight considered dimensions, and the regression coefficient indicating the contribution of satisfaction with health to the overall assessment of QoL was the highest among the eight dimensions [17]. The present study also examines how the subjective importance ratings are related to the importance scores in terms of the dimensions’ contributions to overall HRQoL.

Does domain importance moderate the relationship between domain satisfaction and overall satisfaction? Tiefenbach and Kohlbacher [18] developed and tested the “domain-importance-as-a-moderator” hypothesis. According to this hypothesis, highly important domains should contribute more strongly to overall QoL than less important domains do. The final aim of our study was to test this domain-importance-as a-moderator effect for different aspects of HRQoL.

Taken together, the aims of this study were (1) to investigate the importance of and satisfaction with domains of HRQoL in a large sample of participants enrolled in a cancer rehabilitation program, (2) to analyze the mutual relationship between importance and satisfaction including sex and age differences, (3) to test the relevance of different facets of HRQoL for the prediction of general HRQoL, and (4) to test the domain-importance-as-a-moderator hypothesis for the dimensions of HRQoL.

## 2. Materials and Methods

### 2.1. Sample of Cancer Patients

The study participants were recruited in the oncological rehabilitation clinic in Bad Oexen, Germany, between September 2020 and May 2021. In Germany, cancer patients are generally offered the opportunity to participate in a rehabilitation program to help restore their physical and psychosocial functioning upon cancer treatment completion. During the rehabilitation program, patients receive a variety of treatments tailored to their specific individual needs, including exercises for physical fitness, physiotherapy, relaxation training, and psychological interventions to enhance coping strategies and reduce distress, as well as individual or group counseling that addresses vocational and healthy lifestyle issues.

Inclusion criteria for this study were as follows: proven cancer diagnosis, age 18 years and above, sufficient command of the German language, and absence of severe cognitive impairment. The Ethics Committee of the University of Leipzig approved the study. Informed consent was obtained from the participants after they were given a full explanation of the purpose and nature of the data collection and storage. A total of 1547 consecutive patients were asked to participate, and 1108 (71.6%) of them agreed to take part in the study and to complete the questionnaires during their stay in the rehabilitation clinic.

### 2.2. Instruments

Questions on HRQoL: Since there was no suitable questionnaire available for assessing the subjective importance of HRQoL dimensions, we defined eight dimensions of HRQoL, based on the scales of other relevant questionnaires such as EORTC QLQ-C30 [19] and SF-36 [20]: physical functioning, autonomy, emotional stability, cognitive functioning, social relationships, vitality, absence of pain, and sleep quality. In addition to these specific aspects, the participants were asked to assess their general HRQoL in terms of its importance to them and their satisfaction with it. The first five of the eight dimensions were adopted from the functioning scales of EORTC QLQ-C30. Vitality was taken from SF-36 and can be considered the opposite of fatigue, which is also a scale of EORTC QLQ-C30. Pain is also a component of EORTC QLQ-C30 and SF-36. In contrast to SF-36, we prefer to use the term “absence of pain” instead of “pain” since high scores should represent high degrees of HRQoL for all scales. Sleep quality is also an element of EORTC QLQ-C30 but not SF-36. We included this dimension because of its special relevance for cancer patients [21,22].

Each of these nine dimensions (eight specific dimensions and one general dimension) had to be evaluated concerning two perspectives: “How important is (e.g., physical functioning) for you?”, and “How satisfied are you with your (e.g., physical functioning)?”. Each question could be answered with one of five possible responses: “How important is …”: (not important, …, very important), and “How satisfied are you with your …”: (very dissatisfied, …, very satisfied).

EORTC QLQ-C30: The quality of life questionnaire EORTC QLQ-C30 [19] is the most frequently used HRQoL questionnaire in cancer clinical trials [23]. It consists of 30 items and includes five functioning scales, three symptom scales, six single-item scales, and a two-item global health/HRQoL scale. In our study, we only used this global health/HRQoL scale.

### 2.3. Statistical Analysis

Importance and satisfaction assessments are presented in terms of mean scores and standard deviations. The associations between the importance and satisfaction ratings were calculated using Pearson correlations in accordance with most of the literature on importance and satisfaction analyses. To assess the robustness of the associations, we also calculated Spearman rank correlations.

Age and sex differences were expressed with effect sizes according to Cohen [24]. Since the age distribution was not identical for males and females, we calculated sex differences separately for each age category and averaged these coefficients over the three age groups to quantify the effect size of the sex effect.

Two types of regression analyses were performed using the 2-item global health/QoL scale of EORTC QLQ-C30 as the dependent variable. First, the impact of single satisfaction ratings on this global health/QoL scale was tested with univariate regression analyses. Second, to test the domain-importance-as-a moderator hypothesis for a specific domain, we used the independent variables: satisfaction, importance (dichotomized according to [18]), and their interaction. The effects of age group and sex on importance and satisfaction ratings were statistically tested with 2-way ANOVAs. All statistics were performed with SPSS, version 27.

## 3. Results

### 3.1. Sample Characteristics

Of the 1547 eligible patients, the questionnaire was filled in by 1108 patients—404 males and 704 females—with a mean age of 53.1 ± 14.6 years (range: 18–88 years). Further details of the sample are given in Table 1.

### 3.2. Importance and Satisfaction Ratings

**Figure 1:**
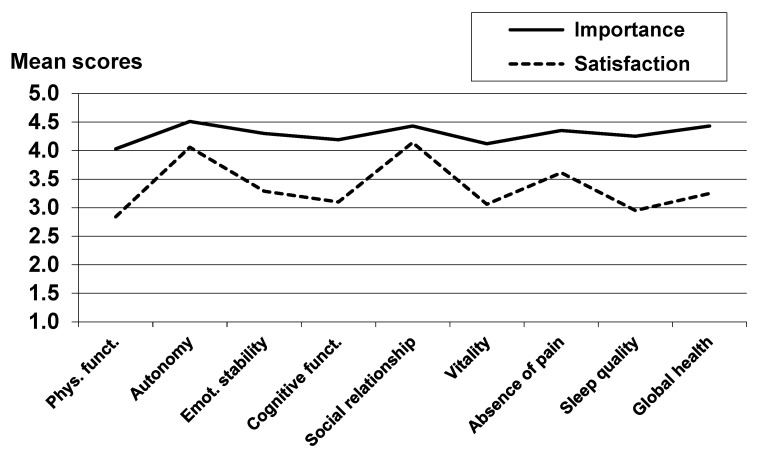
Mean scores of importance and satisfaction ratings.

Table 2 and Figure 1 present mean scores of the importance and the satisfaction ratings. The specific domains with the highest importance ratings were autonomy, social relationships, and absence of pain, while the lowest importance was attributed to physical functioning. Concerning satisfaction, the patients were most satisfied with social relationships and autonomy, whereas the lowest satisfaction ratings were observed for physical functioning and sleep quality.

The Pearson correlations between the importance and the corresponding satisfaction ratings ranged between −0.06 and 0.40 (Table 2), and the global health importance and satisfaction ratings were nearly independent of one another (*r* = 0.01). Using Spearman correlations, the results were very similar, as no difference between the two types of correlations exceeded 0.03.

### 3.3. Sex and Age Differences in Importance and Satisfaction Ratings

The effects of sex and age on importance and satisfaction ratings are presented in Table 3. Females rated social relationships and vitality as being more important than males did (effect sizes *d* = 0.21 and *d* = 0.19), but there were nearly no sex differences in the ratings of the importance of health in general (*d* = 0.03). The older age group attributed less importance to all domains in comparison with the youngest group, with the most pronounced difference in autonomy (*d* = −0.70) and also a remarkable difference (*d* = −0.32) in global health.

Regarding satisfaction, females were less satisfied than males in six of the eight dimensions, in particular in the area of cognitive functioning (*d* = −0.36). Older patients, compared with the youngest age group, were more satisfied with their sleep quality and cognitive functioning, while they were less satisfied than the younger patients with their degree of autonomy. The right column of Table 3 shows that all 16 effects of the interaction between sex and age failed to become statistically significant.

### 3.4. Relationship between Domain Importance, Domain Satisfaction, and Global Health/QoL

Table 4 presents the results of two regression models, both of which used the global health/QoL scale of EORTC QLQ-C30 as the dependent variable. In Model 1, satisfaction was the only independent variable, and in model 2, the independent variables were satisfaction, importance, and their interaction.

According to Model 1, the highest association between global HRQoL and the eight single components of HRQoL was found for vitality (*β* = 0.604), and the lowest association was obtained for social relationships (*β* = 0.318). In Model 2, which included the importance ratings and their interactions, the *β* coefficients of the satisfaction scores were similar to those of Model 1. Among the eight HRQoL dimensions, no importance rating provided a significant contribution to the variance explanation of global HRQoL, and all interaction terms between importance and satisfaction were statistically insignificant.

## 4. Discussion

While multiple examinations have already studied HRQoL in cancer patients and participants in cancer rehabilitation programs, in particular, the main aim of this study was to incorporate assessments of the subjective importance of those HRQoL dimensions in the analyses. Of the eight single dimensions, autonomy was given the highest priority, while physical functioning and vitality were assessed to be the least important. This reinforces the idea that concepts of HRQoL should also include aspects of autonomy, participation, and independence, and not merely the absence of symptoms, a finding that is in line with the goals of rehabilitation in general [25].

The dimensions with the highest satisfaction ratings were autonomy and social relationships. These two dimensions also received the highest importance ratings, which indicates a certain kind of positive relationship between importance and satisfaction. This relationship is underlined by the positive correlations between importance and satisfaction (*r* = 0.35 and *r* = 0.40) for these two dimensions. However, concerning global health, the correlation between importance and satisfaction was negligible (*r* = 0.01). This is lower than the coefficients obtained in general population studies, which reported coefficients of *r* = 0.23 in the USA [8], *r* = 0.04 in Taiwan [11], *r* = 0.08 in Germany [13], and *r* between 0.18 and 0.24 in a German sample with three waves [14]. In a sample of HIV patients, however, the coefficient (*r* = −0.004) was also negligible [10]. The lower associations between importance and satisfaction in groups of patients as compared with those of the general population might be a result of adaptation processes in patients who prioritize the aspect of health more highly after having experienced a chronic disease. To test this possible effect, it would be useful to assess not only the current state of importance but also to measure changes in the importance of health following diagnosis.

There were only small sex effects in the importance ratings. In a large general population study, women rated health as being marginally more important than males did (*d* = 0.05), and older people (≥61 years) rated their health as being markedly more important to them than younger people (≤40 years) (*d* = 0.81) [13]. Of the eight dimensions analyzed in that general population study, health was the dimension with the greatest age difference. In our study, the age effect was much smaller (*d* = 0.02), meaning that younger cancer patients are more concerned about their health problems than older patients in comparison with their healthy peers. This finding is in line with the general findings that younger cancer patients are much more anxious and depressed than their healthy peers, while the difference in anxiety and depression between older cancer patients and their general population peers is much smaller [26]. In a large sample of older adults (mean age 73.1 years), the ability to perform was rated as being even more important than being healthy [27].

In addition to the direct importance ratings, our study also included *β* coefficients in regression analyses, which indicate the relevance of the specific health domains for the global health assessment. The two approaches—namely, direct importance rating and regression coefficients—yielded relatively different results: Social relationships were rated as being highly important, and vitality was rated as being less important, while the predictive power of the vitality satisfaction ratings was markedly higher than that of social relationships. Such divergences are not uncommon: In a general population study, finances were rated as being subjectively unimportant; nevertheless, satisfaction with finances still turned out to be a good predictor of global happiness [8]. In response shift research, three areas are distinguished: recalibration, reprioritization, and reconceptualization [28]. The term reprioritization seems to indicate a change in the subjectively experienced priority of the dimension. However, the assessments do not include importance assessments, and reprioritization is in fact derived from the factor loadings that indicate the association between the specific factor and the underlying construct. The latter is comparable with the *β* coefficients calculated in our study. This underlines that reprioritization effects obtained in response shift research do not mean that the subjective priority or meaning has changed, but rather that the relative correlative position of the factor has changed vis a vis the other factors.

The domain-importance-as-a-moderator hypothesis was not confirmed. Though it sounds plausible that for people who perceive a dimension as being important to them, their satisfaction with this dimension would have a greater impact on an overarching construct (global health) than it would for people for whom that dimension is unimportant; none of the interaction effects were statistically significant. This is in line with general population studies [8] and adds to the body of knowledge on the subject by showing that the hypothesis cannot be confirmed in a large population of cancer patients either. We do not, however, draw the conclusion that domain importance was unimportant [29]. Although these importance ratings do not contribute to a higher variance explanation of a general construct such as HRQoL, the subjective meaningfulness of components of HRQoL should be taken into account by health care providers. While there is an overwhelming body of HRQoL research in oncology that identifies impairments in HRQoL in certain dimensions, the subjective relevance of these dimensions remains largely unexplored. Questions of subjective importance of HRQoL dimensions are not just important in oncology but in all severe chronic diseases. In palliative medicine, in particular, the questions of subjective importance of HRQoL domains arise with particular relevance [30,31,32]. Our study analyzed the subjective importance of HRQoL dimensions as reported by the patients. Physicians may rate the importance of dimensions differently than patients [33,34,35], and practitioners should be aware that their patients may not have the same priorities they assume they would.

Some strengths and limitations of this study should be mentioned. Strengths include the large sample, which allowed separate analysis of subgroups by age and gender, and the equality of the HRQoL domains for determining importance and satisfaction, which allowed clear reference to judgments regarding importance and satisfaction.

One limitation is that the sample is not representative of all cancer patients. Patients who are in good health and therefore decide against taking part in a rehabilitation program may be underrepresented. On the other hand, patients who are highly distressed may not feel up to taking part in such a program.

The instrument for assessing importance and satisfaction is new since there was no suitable instrument available. Each dimension is represented by only one item; assessment instruments with more items per dimension might have provided more reliable assessments. However, in most studies dealing with the subjective importance of domains, single-item measures are used, and these single-item approaches proved to be useful, even in the assessment of complex constructs such as well-being [36]. Moreover, in our study, all dimensions were assessed with single items in a uniform way; therefore, the degree of reliability is similar for the assessments, and the effects of different dimensions can consequently be compared fairly with one another.

Using mean scores, Pearson correlations, and linear regression models is based on the assumption that the variables are of metric character—an assumption that can be doubted. However, for reasons of uniformity and comparability with other studies on this topic, we preferred to use these metric statistics.

We did not specify the results according to the tumor types. Different tumor types may lead to different sequences in the importance ratings of HRQoL dimensions [37]. The association between the importance of health and satisfaction with health can also be analyzed at the group level. When breast cancer survivors are compared with women of the general population, both the importance of and satisfaction with sex life are rated to be lower in the breast cancer group [38], which means that dimensions in which detriments are experienced can decrease in importance for the cancer patients. Comparisons between the importance ratings of HRQoL dimensions between patient groups and the general population would be very helpful here. In our study, the patients rated their current satisfaction with eight HRQoL domains and their currently attributed importance of these domains. It would also be interesting to investigate perceived changes in satisfaction and changes in importance; the relationship between change in importance and change in satisfaction may differ from the relationship of the current variables [39].

Taken together, the associations between importance and satisfaction ratings are weak, which means that the subjective importance of an HRQoL dimension cannot be derived from satisfaction ratings. Although importance ratings are not necessary for a precise assessment of overall HRQoL, they do shed new and relevant light on cancer patients’ subjective experiences. Health care providers should incorporate patients’ subjective preferences and importance assessments in decision making. Due to their high subjective importance, the areas of autonomy and social relationships deserve special attention.

## Figures and Tables

**Table 1:** Sociodemographic and clinical characteristics of the sample (*n* = 1108).

Sociodemographic and Clinical Variables	*n*	%
Sex		
Males	404	36.5
Females	704	63.5
Age group		
18–39 years	220	19.9
40–49 years	183	16.5
50–59 years	327	29.5
60–69 years	233	21.0
≥70 years	145	13.1
Education ^a^		
Elementary school (8–9 years)	248	22.4
Junior high school (10 years)	367	33.2
High school/university (≥11 years)	486	44.0
No formal qualification	4	0.4
Employment status ^a^		
Employed	703	63.7
Unemployed	43	3.9
Retired	282	25.5
Other	76	6.9
Tumor localization		
Breast	381	34.4
Gastrointestinal tract	171	15.4
Prostate	144	13.0
Hematological	131	11.8
Female genital organs	78	7.0
Thyroid/endocrine glands	36	3.2
Melanoma	26	2.3
Male genital organs	23	2.1
Others	118	10.6
Time since diagnosis ^a^		
<6 month	334	30.2
6 month– < 12 months	374	33.8
≥12 months	399	36.0
Treatment		
Surgery ^a^		
No	121	10.9
Yes	986	89.1
Chemotherapy ^a^		
No	508	46.1
Yes	595	53.9
Radiotherapy ^a^		
No	538	50.2
Yes	534	49.8
Hormone therapy ^a^		
No	780	74.0
Yes	274	26.0
Antibody therapy ^a^		
No	864	82.8
Yes	179	17.2

^a^ Missing data not reported.

**Table 2:** Importance and satisfaction mean scores, and correlations between importance and satisfaction, separately for the domains of HRQoL (*n* = 1108).

HRQoL domain	Importance		Satisfaction		*r* (Importance, Satisfaction)	
	M	(SD)	M	(SD)	*r*	*p*
Physical functioning	4.03	(0.65)	2.84	(1.03)	0.16	<0.001
Autonomy	4.51	(0.63)	4.06	(0.95)	0.35	<0.001
Emotional stability	4.30	(0.58)	3.29	(1.02)	0.09	0.004
Cognitive functioning	4.19	(0.62)	3.10	(1.07)	−0.02	0.524
Social relationship	4.43	(0.63)	4.14	(0.88)	0.40	<0.001
Vitality	4.12	(0.62)	3.06	(0.96)	−0.04	0.190
Absence of pain	4.35	(0.67)	3.61	(1.12)	0.04	0.192
Sleep quality	4.25	(0.62)	2.95	(1.18)	−0.06	0.051
Global health	4.43	(0.57)	3.25	(0.92)	0.01	0.652

**Table 3:** Importance and satisfaction ratings by sex and age group (*n* = 1108).

HRQoL Domain		Males				Females						ANOVA *p* Value		
		≤49 y.	50–59 y.	≥60 y.	All	≤49 y.	50–59 y.	≥60 y.	All	d ^(a)^ (Sex)	d ^(b)^ (Age)	Sex	Age	Sex * Age
*n*		80	127	197	404	323	200	181	704					
Importance														
Physical functioning	M	4.16	4.00	3.88	3.97	4.15	4.05	3.90	4.06	0.03	−0.41	0.619	<0.001	0.833
	(SD)	(0.66)	(0.66)	(0.62)	(0.65)	(0.66)	(0.63)	(0.64)	(0.65)					
Autonomy	M	4.67	4.50	4.26	4.42	4.72	4.56	4.27	4.56	0.07	−0.70	0.353	<0.001	0.832
	(SD)	(0.57)	(0.55)	(0.67)	(0.64)	(0.50)	(0.63)	(0.72)	(0.63)					
Emotional stability	M	4.34	4.32	4.14	4.23	4.44	4.37	4.11	4.34	0.07	−0.45	0.222	<0.001	0.370
	(SD)	(0.62)	(0.56)	(0.59)	(0.59)	(0.55)	(0.54)	(0.59)	(0.58)					
Cognitive functioning	M	4.31	4.22	4.13	4.20	4.24	4.20	4.06	4.18	−0.09	−0.29	−0.192	0.001	0.786
	(SD)	(0.61)	(0.62)	(0.62)	(0.62)	(0.64)	(0.61)	(0.60)	(0.63)					
Social relationships	M	4.44	4.32	4.30	4.33	4.55	4.43	4.47	4.49	0.21	−0.17	0.002	0.053	0.711
	(SD)	(0.65)	(0.69)	(0.65)	(0.67)	(0.59)	(0.62)	(0.60)	(0.60)					
Vitality	M	4.16	4.05	3.92	4.01	4.24	4.24	4.00	4.18	0.19	−0.39	0.004	<0.001	0.441
	(SD)	(0.60)	(0.60)	(0.65)	(0.63)	(0.59)	(0.58)	(0.61)	(0.60)					
Absence of pain	M	4.29	4.25	4.30	4.28	4.43	4.44	4.26	4.39	0.14	−0.12	0.029	0.302	0.084
	(SD)	(0.77)	(0.67)	(0.64)	(0.67)	(0.66)	(0.63)	(0.72)	(0.67)					
Sleep quality	M	4.32	4.19	4.21	4.23	4.31	4.26	4.17	4.26	0.01	−0.19	0.946	0.040	0.569
	(SD)	(0.69)	(0.55)	(0.61)	(0.61)	(0.63)	(0.61)	(0.65)	(0.63)					
Global health	M	4.47	4.45	4.32	4.39	4.53	4.44	4.32	4.45	0.03	−0.32	0.663	<0.001	0.737
	(SD)	(0.59)	(0.55)	(0.52)	(0.55)	(0.54)	(0.60)	(0.60)	(0.58)					
Satisfaction														
Physical functioning	M	2.79	2.80	2.99	2.89	2.92	2.65	2.82	2.82	−0.06	0.05	0.343	0.062	0.182
	(SD)	(1.02)	(1.09)	(1.04)	(1.06)	(0.99)	(1.00)	(1.04)	(1.01)					
Autonomy	M	4.13	4.03	3.94	4.01	4.25	4.02	3.87	4.09	0.02	−0.31	0.865	0.001	0.466
	(SD)	(0.89)	(0.89)	(0.99)	(0.94)	(0.94)	(1.01)	(0.90)	(0.96)					
Emotional stability	M	3.32	3.35	3.51	3.42	3.21	3.07	3.38	3.21	−0.17	0.18	0.011	0.007	0.552
	(SD)	(0.99)	(0.96)	(0.95)	(0.93)	(1.05)	(1.06)	(0.99)	(1.04)					
Cognitive functioning	M	3.14	3.29	3.53	3.38	2.90	2.80	3.15	2.93	−0.36	0.32	<0.001	<0.001	0.370
	(SD)	(0.94)	(1.09)	(1.01)	(1.03)	(1.07)	(1.07)	(0.99)	(1.05)					
Social relationship	M	3.95	3.96	4.20	4.08	4.15	4.13	4.30	4.18	0.18	0.23	0.007	0.002	0.752
	(SD)	(0.91)	(0.90)	(0.87)	(0.89)	(0.87)	(0.83)	(0.89)	(0.86)					
Vitality	M	3.21	3.08	3.30	3.21	3.07	2.77	3.01	2.97	−0.26	0.01	<0.001	0.004	0.505
	(SD)	(1.05)	(0.99)	(0.89)	(0.96)	(0.94)	(1.00)	(0.89)	(0.95)					
Absence of pain	M	3.90	3.56	3.82	3.75	3.62	3.34	3.58	3.53	−0.22	−0.06	0.001	0.001	0.957
	(SD)	(1.06)	(1.14)	(0.96)	(1.04)	(1.11)	(1.17)	(1.18)	(1.15)					
Sleep quality	M	2.91	3.13	3.37	3.20	2.74	2.63	3.09	2.80	−0.27	0.35	<0.001	<0.001	0.238
	(SD)	(1.19)	(1.14)	(1.18)	(1.18)	(1.13)	(1.14)	(1.18)	(1.16)					
Global health	M	3.38	3.15	3.42	3.33	3.29	3.02	3.28	3.21	−0.13	0.02	0.041	<0.001	0.952
	(SD)	(0.86)	(1.01)	(0.92)	(0.94)	(0.88)	(0.94)	(0.91)	(0.91)					

^(a)^ Positive effect sizes indicate higher scores for females; ^(b)^ Positive effect sizes indicate higher scores for the oldest age group in comparison with the youngest group.

**Table 4:** Regression analyses. Dependent variable: global health/QoL (*n* = 1108).

HRQoL Domain	Model 1: Satisfaction Only			Model 2: Importance, Satisfaction, and Interaction						
	Satisfaction			Importance		Satisfaction		Importance * Satisfaction		
	*β*	*p*	*R* ^2^	*β*	*p*	*β*	*p*	*β*	*p*	*R* ^2^
Physical functioning	0.530	<0.001	0.281	0.010	0.803	0.533	<0.001	−0.002	0.961	0.286
Autonomy	0.494	<0.001	0.244	−0.004	0.945	0.529	<0.001	−0.066	0.265	0.248
Emotional stability	0.509	<0.001	0.259	0.080	0.125	0.518	<0.001	−0.051	0.349	0.261
Cognitive functioning	0.485	<0.001	0.235	0.062	0.171	0.493	<0.001	−0.052	0.268	0.230
Social relationship	0.318	<0.001	0.101	0.011	0.861	0.335	<0.001	−0.041	0.548	0.102
Vitality	0.604	<0.001	0.365	0.048	0.223	0.612	<0.001	−0.025	0.540	0.368
Absence of pain	0.520	<0.001	0.271	0.068	0.174	0.533	<0.001	−0.038	0.488	0.274
Sleep quality	0.529	<0.001	0.280	−0.001	0.975	0.542	<0.001	−0.030	0.521	0.285
Global health	0.662	<0.001	0.439	0.038	0.492	0.674	<0.001	−0.034	0.552	0.442

## Data Availability

The data presented in this study are available on request from the corresponding author.
